# A modern multi-omics data exploration experience with Panomicon

**DOI:** 10.1093/bioadv/vbae147

**Published:** 2024-10-03

**Authors:** Rodolfo S Allendes Osorio, Yuji Kosugi, Johan T Nyström-Persson, Kenji Mizuguchi, Yayoi Natsume-Kitatani

**Affiliations:** Premium Research Institute for Human Metaverse Medicine (WPI-PRIMe), Osaka University, Suita, Osaka 565-0871, Japan; AI Center for Health and Biomedical Research (ArCHER), National Institutes of Biomedical Innovation, Health and Nutrition, Setsu, Osaka 566-0002, Japan; Lifematics Ltd., Chiyoda ku, Tokyo 101-0051, Japan; JNP Solutions, Sumida, Tokyo 130-0015, Japan; AI Center for Health and Biomedical Research (ArCHER), National Institutes of Biomedical Innovation, Health and Nutrition, Setsu, Osaka 566-0002, Japan; Institute for Protein Research, Osaka University, Suita, Osaka 565-0871, Japan; AI Center for Health and Biomedical Research (ArCHER), National Institutes of Biomedical Innovation, Health and Nutrition, Setsu, Osaka 566-0002, Japan; Institute for Protein Research, Osaka University, Suita, Osaka 565-0871, Japan; Institute of Advanced Medical Sciences, Tokushima University, Tokushima 770-8503, Japan

## Abstract

**Summary:**

To address the challenges of the storage, sharing, and analysis of multi-omics data, here we introduce the newest version of Panomicon, which includes the improvement of the underlying data model, the introduction of new registration and control access service, together with the seamless integration with other services (like TargetMine for data enrichment analysis), integrated in a completely new, more user friendly web application.

**Availability and implementation:**

Panomicon is available online at https://panomicon.nibiohn.go.jp. Unregistered users can access the publicly available data uploaded to Panomicon using the following account: user: guest, password: anonymous. Source code for the application is also freely available under a GNU license at https://github.com/Toxygates/Panomicon/. A brief user guide for the new features of Panomicon is provided as supplementary material online.

## 1 Introduction

The analysis of multi-omics data over the last decade has provided valuable insight in biology and medicine. We can acknowledge that the development of health and precision medicine have in turn created greater needs for multi-omics analysis (Chen *et al.* 2012, Liu *et al.* 2022, Athieniti and Spyrou 2023, Babu and Snyder 2023).

The definition of new methods and tools is, however, not free of challenges, be it either from those that are yet to be completely solved, or those that arise cyclically as part of the constant advancement in science. Accordingly, the development and release of software tools that can deal with this type of dataset has also accelerated (Krassowski *et al.* 2020). Providing a detailed review of the state of the art in multi-omics tool development is outside the scope of this article; for more insight on this, we refer the reader to the special issues on tools for multi-omics data analysis (Cominetti *et al.* 2023) or to the specific reviews on computational methods for oncology data interpretation (Wysocka *et al.* 2023) and drug repurposing (Ahmed *et al.* 2023).

Panomicon (Allendes Osorio *et al.* 2020) is part of this trend of software development specifically tailored for multi-omics. Originally developed as an effort to ease users’ sharing and analysis of general multi-omics datasets, Panomicon was implemented as a web application, with tools that allowed users the upload and analysis of their own datasets, with a special focus on the generation and handling of molecular networks.

Panomicon (Allendes Osorio *et al.* 2020) remains one of the primary access points for the processed, analysis-ready datasets from Open TG-GATEs database (Igarashi *et al.* 2014), together with other publicly available datasets used as supplementary data for previous publications (Allendes Osorio *et al.* 2020).

In addition, the MIT, free license used for the publication of the software, entitles third-party users to run their own instances of Panomicon, and use them to share their own personal databases.

Since its release, Panomicon has been continually maintained and improved. Here, we introduce the latest changes to the tool, which we believe will make Panomicon more accessible for current users, as well as broaden its appeal for a wider audience.

## 2 New in Panomicon

Since its inception, Panomicon has provided a suite of tools aimed at facilitating the thorough exploration of multi-omics data. Through the navigation of different screens that define a single workflow, users are allowed to inspect and generate different views of their multi-omics dataset, which can in turn be saved for analysis elsewhere.

The new version of Panomicon keeps the single workflow approach that we previously introduced. However, several major improvements have been made, including reviewed definitions for the underlying handling of data and a completely revamped user interface. We describe them below. Also, by seamlessly integrating Panomicon with other publicly available tools, it is possible to provide an integrated analysis platform for multi-omics data.

### 2.1 Generalized data model

The data model used for the original version of Panomicon relied on a strictly defined structure of data. Prior to upload, users were required to make sure their data to be structured in terms of exposure time, dose level and compound name. This directly impacted the use of Panomicon as it forced users to comply with a model that did not necessarily fit their original data, and that forcibly imposed an intermediate mapping between the original structure, and the structure that could be handled by Panomicon.

As a tool targeting the flexible environment of multi-omics data, this version of Panomicon implements a more general model that identifies “treatment groups” and corresponding “control treatments” for fold-value computation. This allows us to keep some of the original terminology used in Panomicon, which we think will appeal to more veteran users of the tool; but at the same time introduces a whole new level of abstraction for the way in which data are described and uploaded to Panomicon.

This means that Panomicon now makes very few assumptions about data structure, making it a better fit for heterogeneous omics datasets; whilst at the same time making it easier for users to meet the requirements on data structure.

### 2.2 Migration to Angular

**Figure 1.**
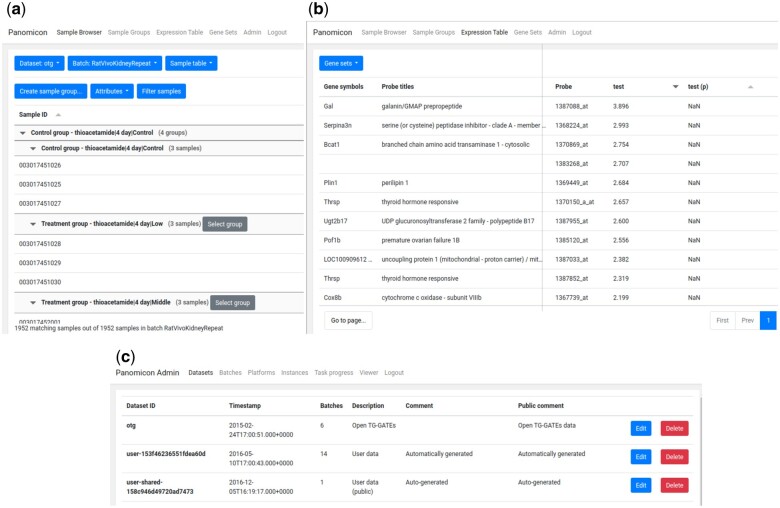
Panomicon’s user interface. The newly implemented interface of Panomicon using the Angular framework. Shown are the Sample Browser (a), Expression Table (b), and Dataset administration (c) tabs.

The original version of Panomicon was implemented using the Google Web Toolkit (https://www.gwtproject.org) as an application framework. While that was an appropriate technology at the time, it no longer provides a modern experience. In order to provide a better experience for our current user base, and a smoother learning curve for future users, we have converted core parts of the codebase into a JavaScript application based on the Angular (https://angular.io/) framework. The result of this migration is shown in Fig. 1. We believe that this will provide a stable foundation for the future development of Panomicon. We will also continue to provide the original Panomicon application for the foreseeable future.

### 2.3 Access control

Although the reproducibility of multi-omics analysis is largely based on the public availability of both tools and data; sometimes data access needs to be restricted to a specific audience. Previously, users were not able to include any restrictions on the access to data once it had been uploaded to Panomicon, which in turn deterred potential users from using this service.

In the latest release of Panomicon, we require that users are registered in order to use the application. This is implemented using the FusionAuth (https://fusionauth.io/) framework. Granular access control allows system administrators to deny or grant access to datasets on an individual basis. This is not intended as a complete information security solution for highly sensitive data but should be a practical technology in many cases.

### 2.4 Sample search based on arbitrary conditions

The generalized data model is combined with a new GUI that allows users to define filters using Boolean conditions such as “liver weight > 10 g AND body weight > 240 g.” Multiple attributes can be added to the table display via the “Attributes” drop-down menu available in the “Sample Table” user interface. Attribute selection, combined with the definition of filters, allows for easy sample browsing and data discovery.

### 2.5 Statistical summaries

Samples can be counted and summarized in terms of freely selected sample parameters, similar to a “pivot table” in a spreadsheet. This might be used to see e.g. how many samples for each treatment compound and species are present.

As with the “Sample Table” interface, multiple attributes for the current dataset can be added to the display of statistical summaries. This is done by sequentially selecting the “Add attribute” button. Notice that entries need to be regenerated after selecting the attributes of interest.

### 2.6 Improved integration with TargetMine

TargetMine (Chen *et al.* 2022) is a Data Warehouse powered by the InterMine framework (Smith *et al.* 2012) hosted by the National Institute of Biomedical Innovation, Health and Nutrition of Japan. A series of different analysis tools are readily available within TargetMine, including gene enrichment, integrated pathway clusters analysis, composite network generation, among others (Chen *et al.* 2022).

Panomicon can synchronize gene (or any other biomolecule) sets with TargetMine and other instances of the InterMine framework. In this new version, Panomicon lets users select which platform (e.g. RefSeq, microarrays) to import gene sets into, which allows for a simpler and more precise data analysis.

## 3 Discussion

The focus on the development of tools that can ease the storage and analysis of multi-omics datasets remains strong. Here, we have introduced the latest version of Panomicon, a tool that thanks to its new and improved features, constitutes a substantial contribution to this trend.

Panomicon uses a completely revamped model for the internal handling of data. The only assumption that the system now makes is the presence of “treatment” and “control” groups as an abstraction layer for the two groups required for log-fold calculations. The introduction of user-defined metadata files at the loading step introduces a general data model that is much more flexible than the one used in previous versions of the tool.

Panomicon also includes a completely redesigned user interface, which will provide a much more comfortable and easier to use environment for both previous and potential new users.

The new interface is also accompanied by an improved, subject agnostic, underlying data representation method that better adjusts to the heterogeneity of multi-omics datasets.

Also, by integrating Panomicon with services like TargetMine, we can provide an extended analysis platform, with an extensive heterogeneous source of background data.

In addition, and as a complementary effort into making it easier for users to deploy their own versions of Panomicon, we intend to provide the application as a set of Docker images, in addition to the source code available at GitHub.

Overall, we believe that the new version of Panomicon will be seen as an important step forward by current users and will have a better adoption appeal for new users. However, we also believe there is great potential for further development and growth of Panomicon. We currently have future goals around two principal lines: the addition of new inference and analysis methods and the integration of additional external analysis tools with Panomicon.

## Supplementary Material

vbae147_Supplementary_Data

## Data Availability

No new data were generated or analysed in support of this research. Data previously loaded to Panomicon is also available in the Gene Expression Omnibus (GEO), and can be accessed with GSE29250 at https://www.ncbi.nlm.nih.gov/geo/query/acc.cgi?acc=GSE29250.
