# Supplementary material for: A modern multi-omics data exploration experience with Panomicon
Source: Bioinform Adv. 2024 Oct 3;4(1):vbae147. doi: 10.1093/bioadv/vbae147 (PMC11520228; doi:10.1093/bioadv/vbae147)
Supplement: vbae147_Supplementary_Data [file vbae147_supplementary_data.pdf]

Sample discovery and analysis with Panomicon (2023 version)

In this guide, we will walk through a brief example of how to use Panomicon for multi-omics sample discovery and data exploration, based on Open TG-GATEs toxicogenomic data. Panomicon may be accessed at https://panomicon.nibiohn.go.jp.

Registering a user account

A user account is required to use Panomicon. On loading, users are presented with a dialog screen that allows logging-in to a previously created account. Alternatively, it is possible to register a user account using the "create account" button. To set up a user account, an e-mail address is necessary.

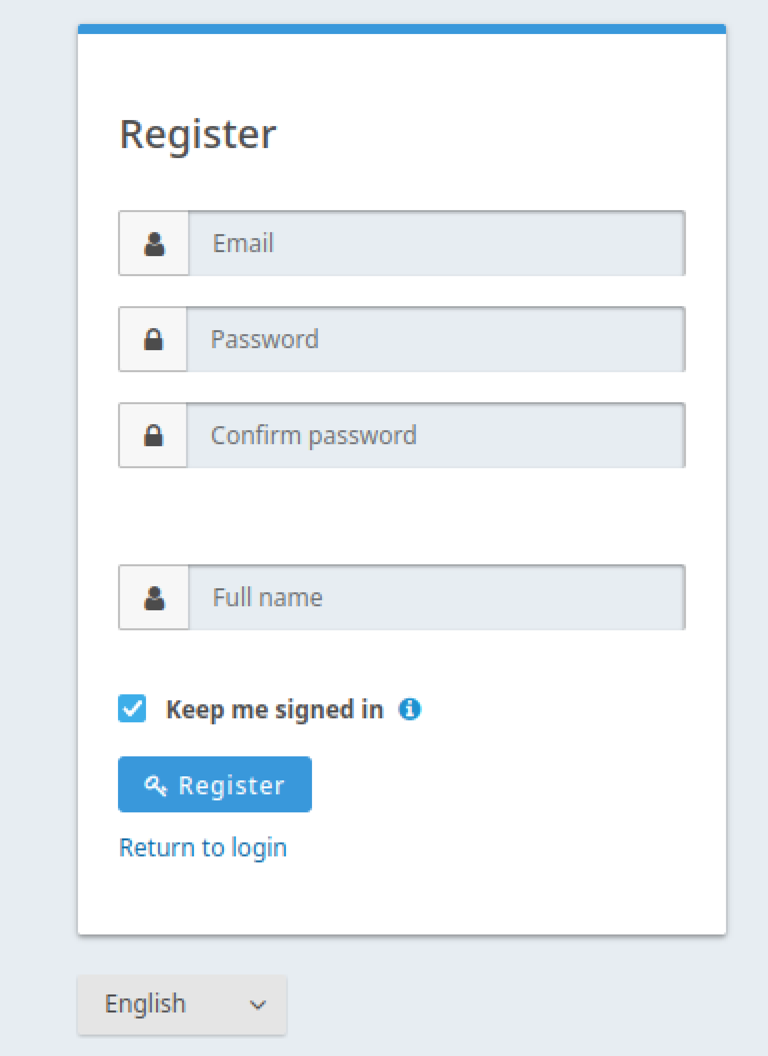

Upon creating a new account (or logging in), the user is immediately forwarded to Panomicon's main interface.

Locating samples

After you have logged in, you can access the Panomicon interface. It is divided into several screens, accessible through the links in the top navigation bar at the top. The default screen is "Sample browser".

Sample treatments

Within the *Sample Browser* interface, the user is able to browse the different datasets included in Panomicon. Currently, by default, only the Open TG-Gates (or *otg*) dataset is available to the general public.

Using the available drop-down list controls, users are able to select different datasets and batches within these datasets. The following figure shows an example of how the data is actually displayed upon selection of a specific batch.

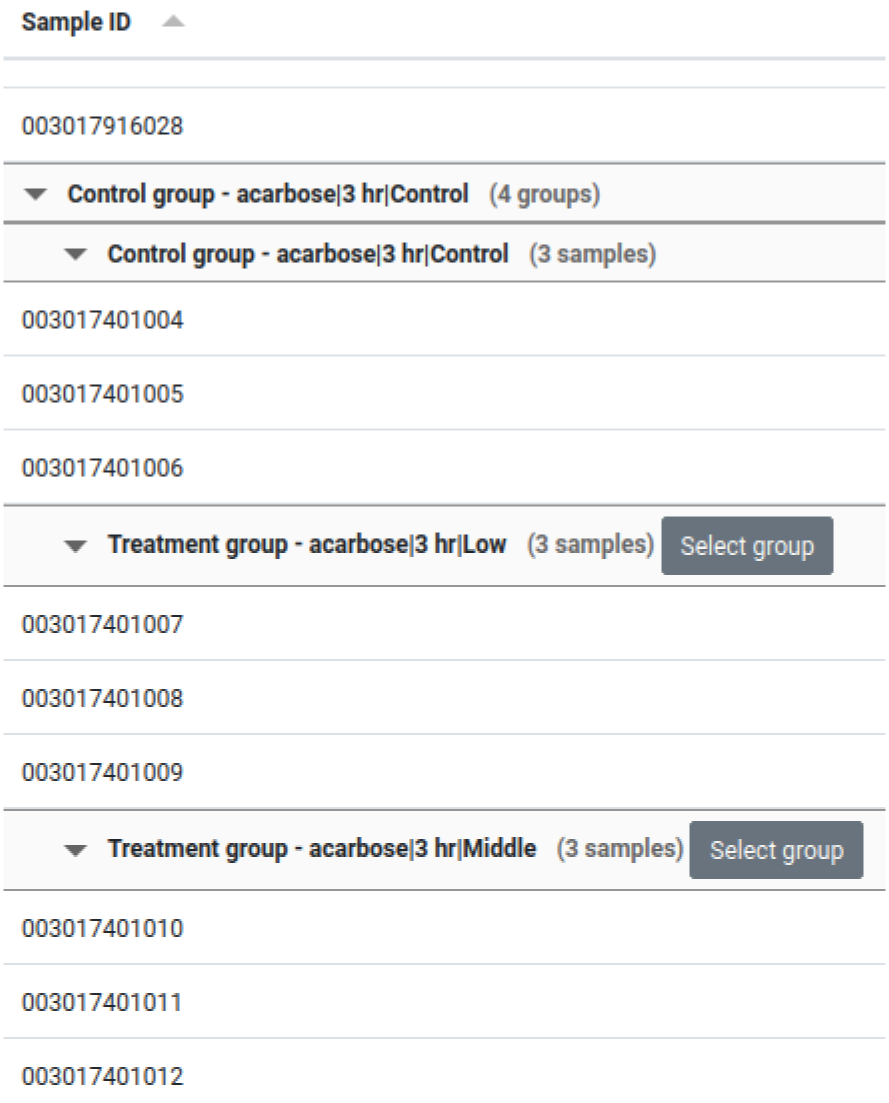

Selecting visible attributes

As shown in the previous image, initially only sample IDs are included in the table display. Additional (sample attributes) can be manually selected for display. This is done in the **Attributes** drop-down menu.

Filtering treatments by attribute values

Custom filters can be defined in order to reduce the number of samples displayed. The following image shows the custom dialog box used to define filters (reached by using the **Edit filters** button).

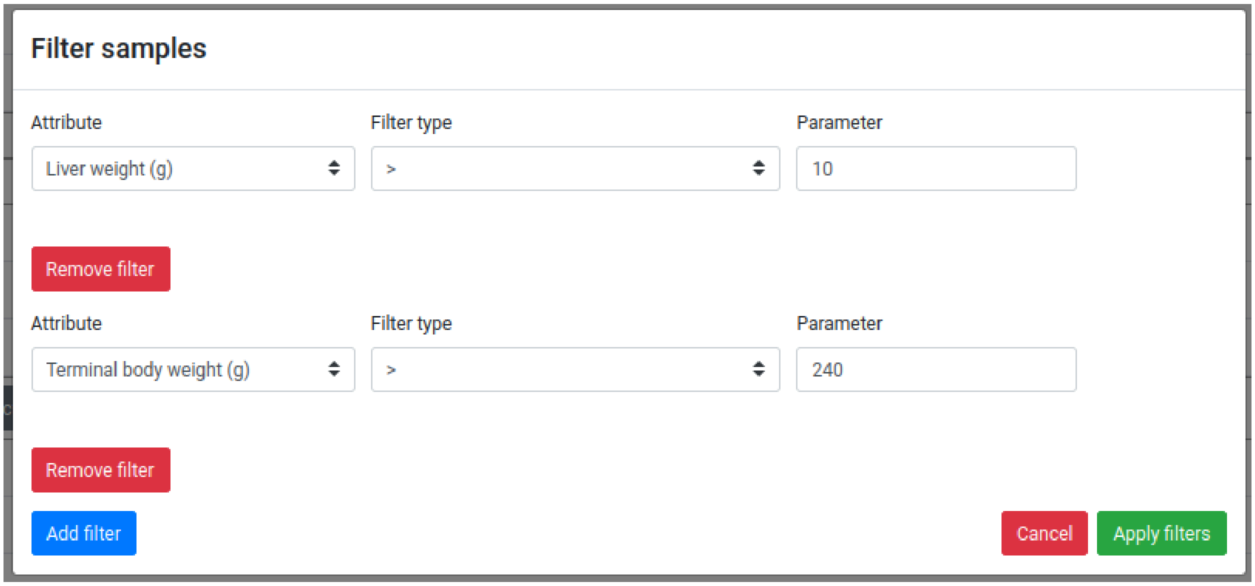

After filters have been applied, only sample treatments that contain at least one sample that passes the filters will be displayed. Numerical values passing a threshold will be shown in blue, while values that do not pass will be shown in red.

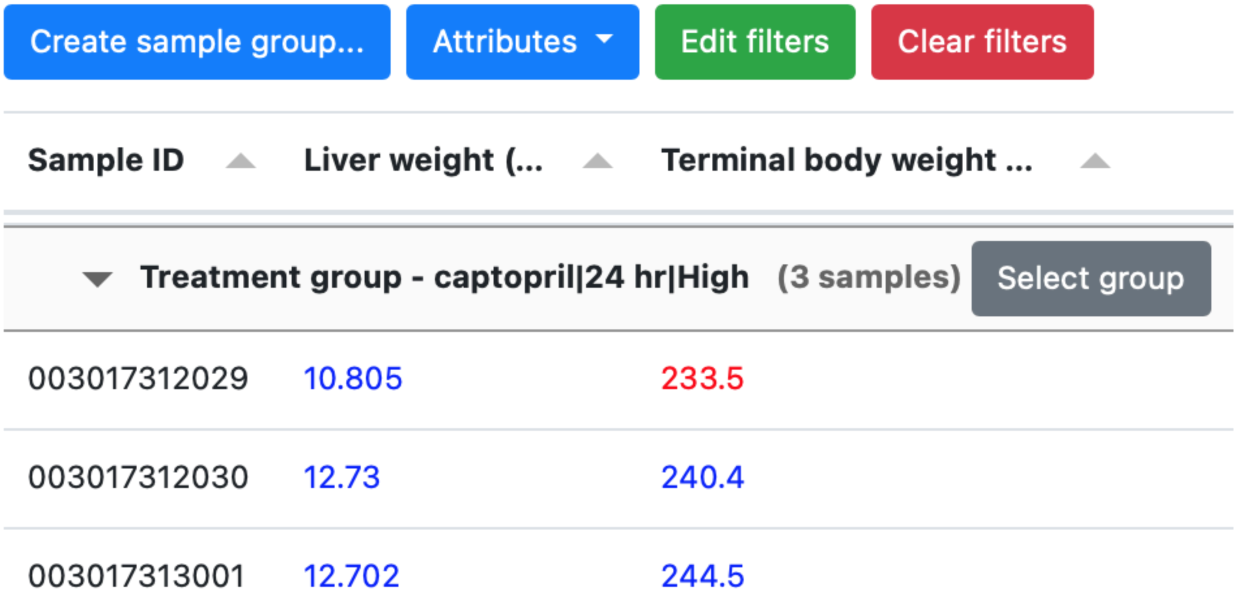

Statistical overview of samples

In the dropdown box initially labeled "sample table", it is possible to select "batch statistics". This displays a different interface.

Selecting different attributes

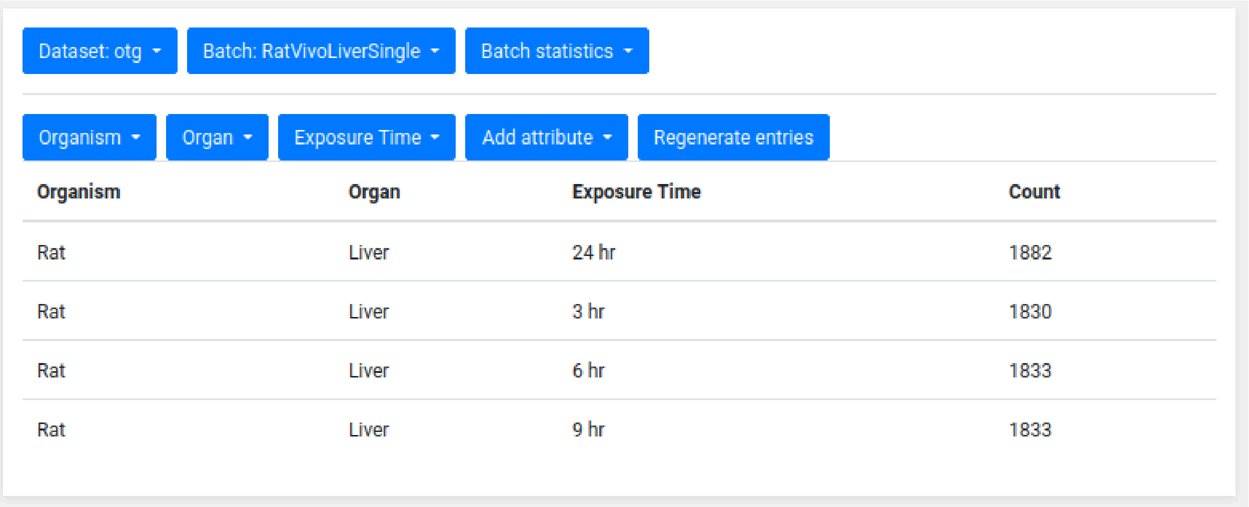

Organism	Organ	Exposure Time	Count
Rat	Liver	24 hr	1882
Rat	Liver	3 hr	1830
Rat	Liver	6 hr	1833
Rat	Liver	9 hr	1833

Defining sample groups

To view gene expression data, it is necessary to define at least one sample group. Sample groups are defined by selecting treatment groups using the **Select group** buttons next to treated samples. After at least one treatment group has been selected in this way, the group may be saved using the **Create sample group** button. The process may be repeated to create multiple groups.

Saved groups are persisted in the web browser and may be viewed, renamed, enabled/disabled, and deleted on the **Sample groups** screen.

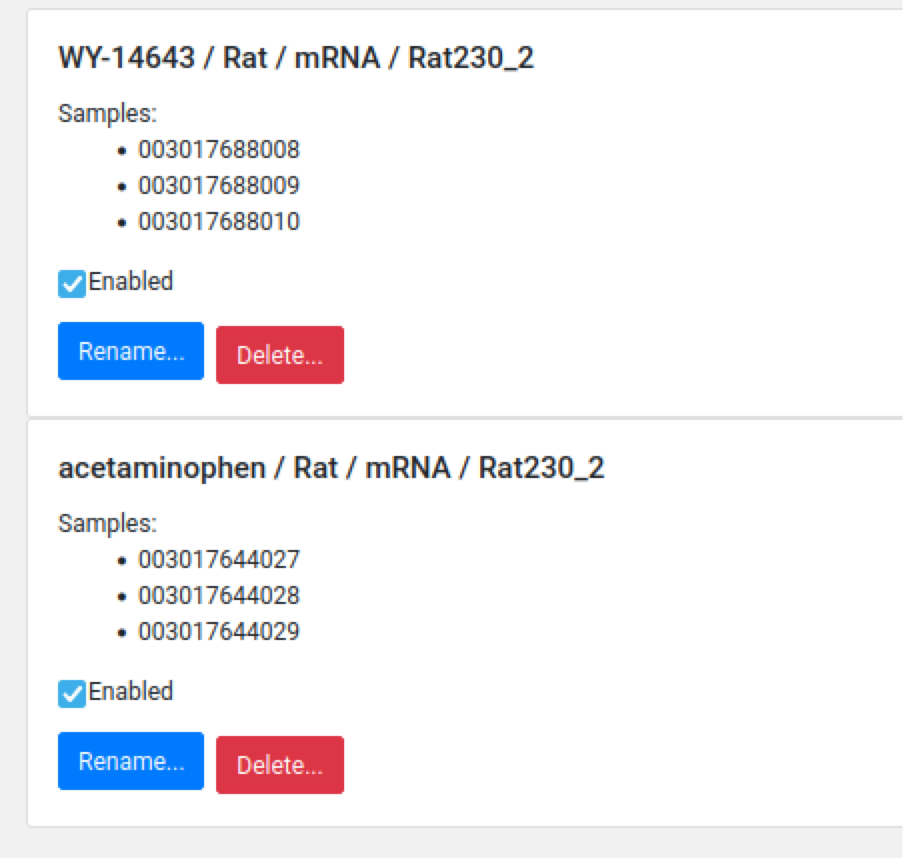

Viewing gene expression data

Once at least one sample group has been defined, data may be viewed on the "Expression table" screen.

Gene symbols	Probe titles	Probe	WY-14643	WY-14643 (p)
Acot1	acyl-CoA thioesterase 1	1398250_at	5.326	0.2178
Abhd3	abhydrolase domain containing 3	1382137_at	4.918	0.2462
Crot	carnitine O-octanoyltransferase	1380015_at	3.789	0.2367
Acot3	acyl-CoA thioesterase 3	1378169_at	3.108	0.1336
Pdk4	pyruvate dehydrogenase kinase - isozyme 4	1378074_at	3.092	0.0005
Mlc1	megalencephalic leukoencephalopathy with subcortical cysts 1	1376702_at	2.964	9.055e-5

Other functions

A complete introduction to all the functions of Panomicon is beyond the scope of this document. Additional functions include gene set management and TargetMine connectivity, among others. We encourage users to explore the tool for themselves based on the concepts introduced in this guide.
